# Note from the Editor: Addressing Applications of Genomics Data

**Published:** 2004-08

**Authors:** Thomas J. Goehl

**Affiliations:** Editor-in-Chief, *EHP,* NIEHS, National Institutes of Health, Department of Health and Human Services, Research Triangle Park, North Carolina, E-mail: goehl@niehs.nih.gov

The two editorials in this issue of *EHP* address the application of toxicogenomics data in the risk assessment and regulatory processes. Also addressing these issues is the National Research Council/National Academy of Sciences Committee on Emerging Issues and Data on Environmental Contaminants. (http://dels.nas.edu/emergingissues/index.asp). This committee, formed at the request of the National Institute of Environmental Health Sciences (NIEHS), provides a public forum for discussing emerging issues in environmental toxicology.

The committee comprises experts from academia, industry, and public interest groups whose specialties include toxicology, toxicogenomics, genetics, bioinformatics, risk assessment, medical ethics, epidemiology, communications, public health. In addition, a U.S. federal government liaison group has been created to work with the committee with representatives from the NIEHS, the Centers for Disease Control and Prevention, the Environmental Protection Agency, the Food and Drug Administration, the Department of Agriculture, the Department of Energy, the Occupational Safety and Health Administration, the Department of Defense, and the Department of Transportation.

A subcommittee is being formed to write a “Consensus Report on the Applications of Toxicogenomic Technologies to Predictive Toxicology.” This report will highlight how the study of gene and protein activities and other biological processes can improve the characterization of toxic substances and their potential risks. Ultimately, this report should show how major new or anticipated uses of these technologies could improve the protection of public health and the environment.

